# Tapioca Melanoma of the Iris: A Case Report

**DOI:** 10.18502/jovr.v14i3.4794

**Published:** 2019-07-18

**Authors:** Saeed Karimi, Pouyan Pahlevani

**Affiliations:** ^1^Ophthalmic Research Center, Shahid Beheshti University of Medical Sciences, Tehran, Iran; ^2^Department of Ophthalmology, Torfeh Medical Center, Shahid Beheshti University of Medical Sciences, Tehran, Iran

**Keywords:** Brachytherapy, Iris Melanoma, Tapioca Melanoma

## Abstract

**Purpose:**

To report an extremely rare case of tapioca melanoma of the iris in an Iranian patient.

**Case Report:**

A 50-year-old male patient presented with ocular pain and redness in the right eye for two weeks. The visual acuity was 7/10 and the intraocular pressure (IOP) was 30 mmHg. A lobulated amelanotic vascularized and nodular (tapioca-like) iris mass with a 180${}^{\text{o}}$ extent was seen in the right eye. Incisional biopsy of the mass revealed atypical mixed type (epithelioid and spindle cell) melanoma. Brachytherapy with the ruthenium-106 plaque resulted in complete regression of the tumor.

**Conclusion:**

Tapioca melanoma of the iris should be considered in the differential diagnosis of patients presenting with nodular vascularized amelanotic iris mass and elevated IOP. Brachytherapy with ruthenium-106 seems to be an effective treatment for tapioca melanoma of the iris.

##  INTRODUCTION

Tapioca melanoma of the iris is a rare form of diffuse iris melanoma,^[1,2]^ typically presenting with heterochromia and elevated intraocular pressure (IOP), mainly because of aqueous outflow blockade. This tumor could also manifest as a diffuse, circumferential, and lightly pigmented or amelanotic lesion.^[3,4]^ Almost 3–5% of patients develop distant metastasis of tumor.^[5]^ Histopathologically, tapioca melanoma is similar to other types of iris melanomas, but can be distinguished by their specific clinical presentations.^[6]^ Tapioca melanoma is characterized by weakly pigmented spindle or epithelioid malignant cells with a spreading pattern.^[3,4]^ Compared to the other types of iris melanomas, tapioca melanoma is an uncommon type characterized by seeding and high IOP in younger patients.^[5]^ A collection of epithelioid cells in the anterior chamber angle may cause glaucoma.^[4]^ The S-100 is a strong marker for diagnosis of cutaneous melanoma, but iris melanomas have diverse morphologies and different abilities to take up this stain. Therefore, diagnosis of atypical iris melanomas may be difficult and challenging.^[2,7]^ Early diagnosis and treatment of atypical iris melanomas are essential for preventing the progression of the disease and saving the patients' eyesight and life.^[8,9]^

##  CASE REPORT

**Figure 1:**
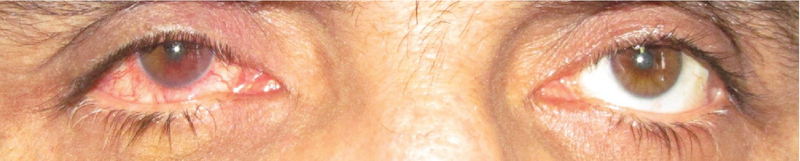
Primary presentation: Conjunctiva and ciliary injection of the right eye.

**Figure 2:**
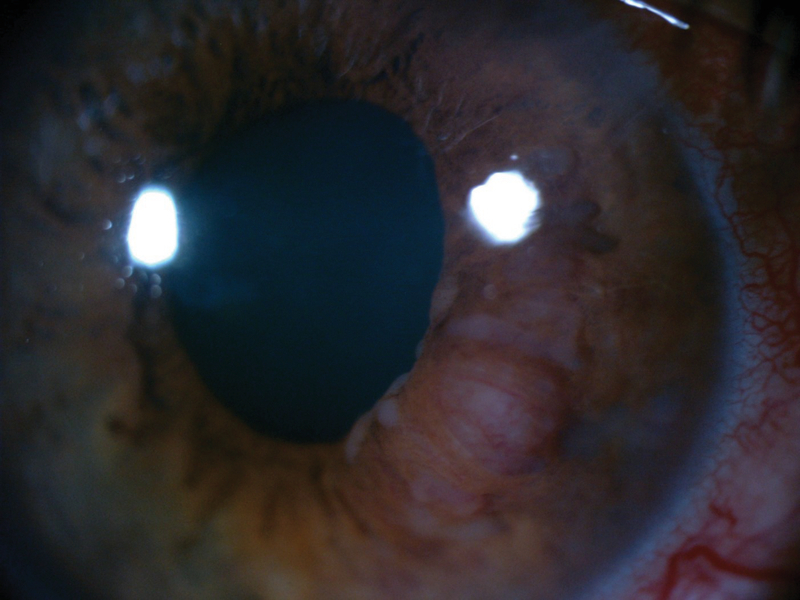
Amelanotic, nodular, and vascularized iris mass with prominent feeder vessels.

**Figure 3:**
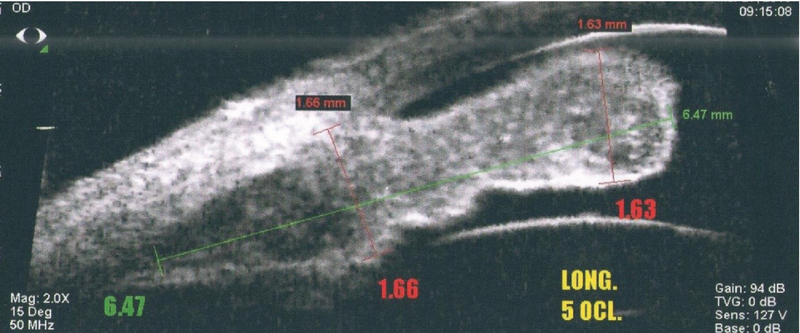
High resolution ultrasound biomicroscope shows diffuse thickening and infiltration of iris with the tumor.

**Figure 4:**
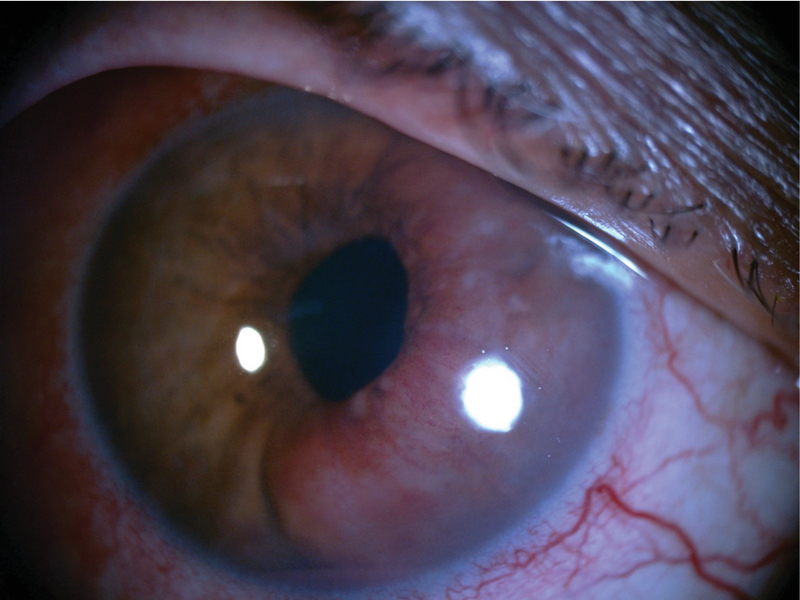
Enlarged vascularized iris mass, two weeks after first visit.

**Figure 5:**
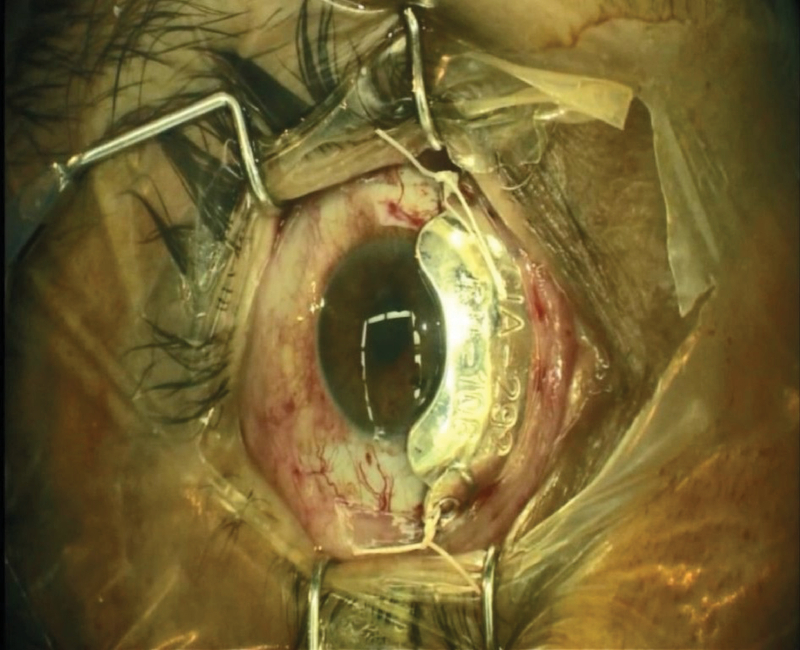
Ru-106 plaque (CIA design) to treat the iris tapioca melanoma.

**Figure 6:**
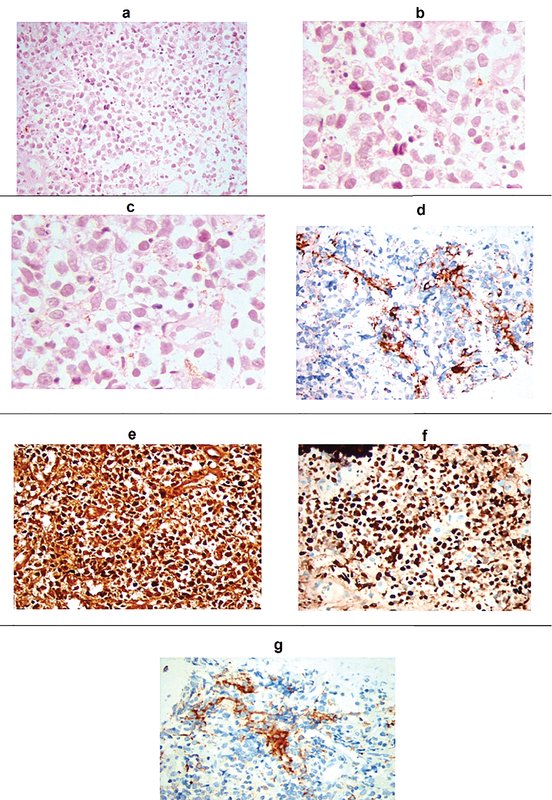
Histopathologic images (a) Epithelioid and spindle-shaped neoplastic cells with apoptotic bodies and mild scattered melanin pigments [hematoxylin and eosin stain (H&E) ×400]; (b,c)-Dysplastic tumor cells mainly of epithelioid type with large nuclei, prominent nucleoli, and apoptosis, H&E ×1000, higher magnification; Patchy immune-reactivity to HMB45 (d), strong immune-reactivity to S100 (e) and Ki67 (f), magnification ×400; (g) Patchy immune-reactivity to melan-A, magnification ×400.

**Figure 7:**
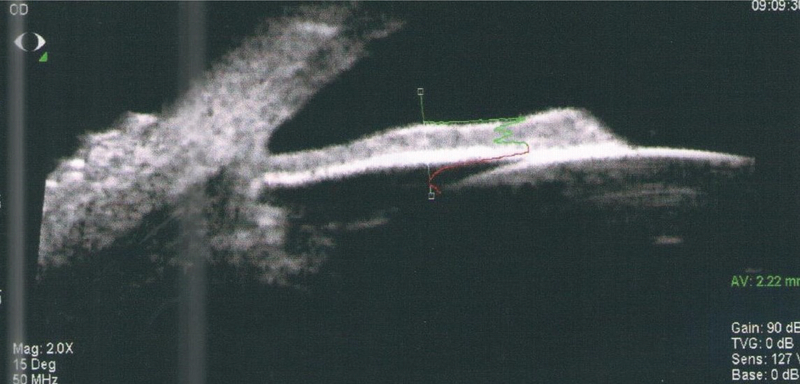
High resolution ultrasound biomicroscopy (UBM) of the iris, five months after brachytherapy with ru-106 plaque reveals complete tumor regression and iris atrophy.

**Figure 8:**
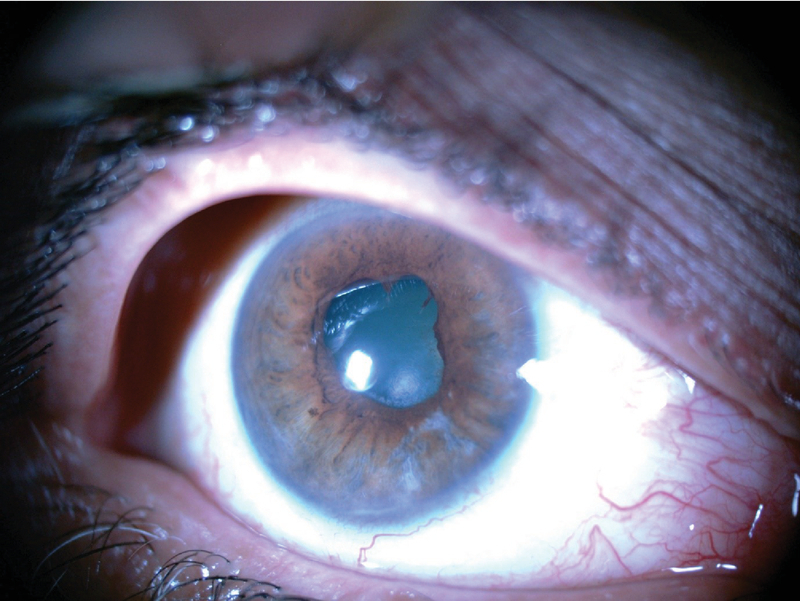
Photo-slit image of the right eye five months after treatment: Complete tumor regression, iris atrophy, localized posterior synechia, and mild cataract noted.

A 50-year-old male patient presented with ocular pain and redness in the right eye for two weeks. The best-corrected visual acuity was 7/10 and 9/10 in the right and left eyes, respectively, without afferent pupillary defect. Extraocular movements were within normal limits in both eyes. Except for controlled diabetes mellitus, the past medical and surgical history were unremarkable. Slit lamp examination revealed “1+" cells in the anterior chamber and fine keratic precipitates on the corneal endothelium. There was a lobulated, nodular, amelanotic, and highly vascularized mass measuring about four clock hours of the iris nasally, disrupting the normal iris structure [Figures 1 and 2]. The IOP was 16 and 15 mmHg in the right and left eyes, respectively. Dilated fundus examination and optical coherence tomography images revealed moderate non-proliferative diabetic retinopathy without diabetic macular edema in both eyes. Ultrasound biomicroscopy (UBM) determined diffuse iris thickening (2.02 mm) and tumoral involvement of the iris with minimal spread to the ciliary body [Figure 3]. Systemic work-up tested negative for metastasis, granulomatous diseases, or any extraocular primary tumor. Two weeks later, ocular pain and redness increased, the IOP peaked at 30 mmHg, and the mass grew and involved the six o'clock region of the iris [Figure 4]. With the provisional diagnosis of iris tapioca melanoma, incisional biopsy of the iris and brachytherapy with ruthenium-106 CIA radioactive plaque (Eckert & Ziegler BEBIG GmbH, 100 Gy, Berlin, Germany) were performed simultaneously [Figure 5]. Microscopic examination of the biopsied tissue demonstrated atypical epithelioid and spindle cells with large nuclei and prominent nucleoli with melanin pigments [Figure 6]. There was a strong immunoreactivity to S-100 and Ki-67 and patchy immunoreactivity to HMB45 and melan-A. All examinations revealed a rare subtype of iris malignant melanoma called tapioca melanoma. No metastasis was found in the systemic work-up. One week after treatment, the tumor regressed, and IOP decreased to 14 mmHg. Five months after brachytherapy, there was no sign of tumor recurrence or metastasis. Slit lamp examination and UBM demonstrated complete tumor regression with iris atrophy and mild lens opacity [Figures 7 and 8].

##  DISCUSSION

Tapioca melanoma, a rare form of diffuse iris melanoma presenting as a hyperchromic iris heterochromia with unilateral elevation of IOP due to the aqueous outflow obstruction.^[4]^ It seems that agglomeration of epithelioid cells in the anterior chamber angle is the cause of IOP elevation.^[3]^ Tapioca is derived from a native Brazilian word denoting the pale granular starch material obtained from manioc tubers.^[6]^ Algernon Reese used the term “tapioca melanoma" for the first time in 1972, owing to the resemblance of these pale tumor nodules to tapioca pudding.^[2,3,10]^ Earlier in 1959, Lorenz E Zimmerman diagnosed this lesion and described it as an atypical iris melanoma.^[3]^ Compared to the other types of iris melanoma, tapioca melanoma mostly occurs in younger patients.^[1]^ The youngest reported case was that of a seven-year-old patient.^[3]^ There could be a connection between the inception of hormonal changes at puberty and the commencement of uveal melanoma.^[11]^ The mean age of surgery in tapioca melanoma patients was 30 years, whereas in other types of iris melanoma it was 46.^[3]^ Our patient was 50 years old, but remarkably, this rare type of melanoma is very uncommon in adults. Different presentations of tapioca iris melanoma in different populations may be related to the varying genomic types of the disease.^[12]^

There are several predisposing factors of uveal melanoma, including ocular melanocytosis, neurofibromatosis type 1, dysplastic nevus syndrome, and familial uveal melanoma.^[11]^ However, there was no remarkable family history for uveal melanoma in this case. This tumor usually presents as a vascularized mass with nodular surface.^[6]^ Diseases such as Fuch's heterochromic iridocyclitis, neurofibromatosis, and Cogan-Reese may present with iris nodules and elevated IOP.^[1]^ In neurofibromatosis, there are yellow-brown dome-shaped papules on the iris called Lisch nodules, while Cogan-Reese presents irregular corneal endothelium with pedunculated and pigmented nodules. In sarcoidosis, patients have systemic signs and symptoms along with papillitis, papilledema, and granulomas in any part of the eye.^[13]^ Juvenile xantogranuloma, Lisch and Koeppe nodules, infectious granulomas, vascular tumors, leiomyoma, metastases, and iris nevus syndrome are some of the relevant differential diagnosis.^[8,12]^ Due to the rarity of tapioca iris melanoma, there are no well-defined criteria for the identification of this type of tumor, and hence its diagnosis can be challenging. Although the iris tumor in our case may not have had the exact appearance of a typical tapioca-like iris melanoma, it could be classified as a tapioca iris melanoma considering its characteristic iris nodules, anterior uveitis, rapid growth, high IOP, and high rate of vascularization. Immunohistochemical staining is often used to confirm the diagnosis. S-100 marker is usually used to stain melanoma and neural tissues, while Ki-67 and HMB-45 are used to mark proliferating cells.^[1,8]^ In the present case, immunostaining was positive for S-100, HMB-45, Ki-67, and melan-A that confirmed the diagnosis of iris melanoma.

There is no agreement on the specific size of iris tumor for surgical treatment. Some authors believe in tumor excision for lesions > 3 mm in diameter or 1 mm in thickness.^[13]^ Sectoral iridectomy is advocated in patients with localized tumors that are limited to the iris without satellite lesions, while en bloc excision is recommended when the anterior chamber angle is involved.^[9]^

Leiden used ruthenium-106 plaque brachytherapy to treat the iris and anterior ciliary body melanomas for the first time in 1997.^[14]^ Only two recurrences were observed among 36 patients with anterior uveal melanoma during the six and a half years of follow-up. Ruthenium-106 plaques are β-emitter sources. Therefore, the optic disc and macula are exposed to lower doses of radiation as compared to the β-emitter sources like I-125 plaques. ^[15]^ Ruthenium-106 plaque treatment for iris melanoma has a significant effect on the control of tumor without any recurrence.^[16]^ Our patient was treated with ruthenium-106 CIA plaque, and there was complete regression of the tumor without any recurrence after 18 months of follow-up.

In conclusion, tapioca iris melanoma should be considered in differential diagnosis of any iris tumor presenting as a nodular vascularized iris mass and elevated IOP.^[1]^ Brachytherapy with ruthenium-106 plaque would be a useful treatment for tapioca iris melanoma.

##  Financial Support and Sponsorship

Nil.

##  Conflict of interest

There are no conflicts of interest.
